# Circulating Interlukin-32 and Altered Blood Pressure Control in Individuals with Metabolic Dysfunction

**DOI:** 10.3390/ijms24087465

**Published:** 2023-04-18

**Authors:** Melissa Tomasi, Alessandro Cherubini, Serena Pelusi, Sara Margarita, Cristiana Bianco, Francesco Malvestiti, Lorenzo Miano, Stefano Romeo, Daniele Prati, Luca Valenti

**Affiliations:** 1 Precision Medicine Lab—Department of Transfusion Medicine, Fondazione IRCCS Ca’ Granda Ospedale Maggiore Policlinico, 20122 Milan, Italyalessandro.cherubini@policlinico.mi.it (A.C.);; 2 Department of Pathophysiology and Transplantation, Università degli Studi di Milano, 20122 Milan, Italy; 3 Department of Molecular and Clinical Medicine, Institute of Medicine, Sahlgrenska Academy, Wallenberg Laboratory, University of Gothenburg, 413 45 Gothenburg, Sweden; 4 Department of Cardiology, Sahlgrenska University Hospital, 413 45 Gothenburg, Sweden; 5 Clinical Nutrition Unit, Department of Medical and Surgical Science, University Magna Graecia, 88100 Catanzaro, Italy

**Keywords:** arterial hypertension, hepatic fat accumulation, IL-32, inflammation, MAFLD, NAFLD

## Abstract

Fatty liver disease is most frequently related to metabolic dysfunction (MAFLD) and associated comorbidities, heightening the risk of cardiovascular disease, and is associated with higher hepatic production of IL32, a cytokine linked with lipotoxicity and endothelial activation. The aim of this study was to examine the relationship between circulating IL32 concentration and blood pressure control in individuals with metabolic dysfunction at high risk of MAFLD. IL32 plasma levels were measured by ELISA in 948 individuals with metabolic dysfunction enrolled in the Liver-Bible-2021 cohort. Higher circulating IL32 levels were independently associated with systolic blood pressure (estimate +0.008 log_10_ per 1 mmHg increase, 95% c.i. 0.002–0.015; *p* = 0.016), and inversely correlated with antihypertensive medications (estimate −0.189, 95% c.i. −0.291–−0.088, *p* = 0.0002). Through multivariable analysis, IL32 levels predicted both systolic blood pressure (estimate 0.746, 95% c.i 0.173–1.318; *p* = 0.010) and impaired blood pressure control (OR 1.22, 95% c.i. 1.09–1.38; *p* = 0.0009) independently of demographic and metabolic confounders and of treatment. This study reveals that circulating IL32 levels are associated with impaired blood pressure control in individuals at risk of cardiovascular disease.

## 1. Introduction

Fatty liver disease affects roughly a quarter of the global population, is most frequently associated with metabolic dysfunction (MAFLD: metabolic dysfunction associated fatty liver disease) and is rapidly becoming a leading cause of advanced liver disease. Accumulation of neutral lipids within intracellular lipid droplets in hepatocytes is the hallmark of MAFLD [1], but progression to severe disease is accompanied by several pathophysiological events triggered by lipototoxicity. This process leads to activation of non-parenchymal cells, including sinusoidal endothelial cells, Kupffer cells and stellate cells, resulting in the infiltration of the liver by inflammatory cells and activation of fibrogenesis [1]. MAFLD is increasingly being recognized as a multisystemic disease, affecting extrahepatic organs and has been associated with arterial hypertension [2,3,4], increasing the risk of cardiovascular events. Although systemic inflammation associated with MAFLD can promote endothelial cells dysfunction [5,6], the role of hepatic fat accumulation in the development of arterial hypertension remains poorly understood [7,8]. 

Arterial hypertension, defined by persistently elevated blood pressure, is a major cardiovascular disease (CVD) risk factor, including heart failure and stroke, peripheral artery disease and chronic kidney disease (CKD). Arterial hypertension is a multifactorial disease, affecting up to 40% of the population and causing 7.7–10.4 million annual deaths worldwide [9,10]. Despite many efforts, hypertension remains one of the most challenging disorders to study and treat due to the complex nature and unknown causes. In recent years, accumulating evidence suggests a correlation between inflammatory mediators (IL-1β, IL-6, IL-8, IL-17, IL-23, TGFβ and TNFα) and elevated blood pressure promoting vascular and endothelial cell dysfunction [11,12,13,14], although the mechanism is not completely defined.

We recently identified Interleukin-32 (IL32) as the most upregulated transcript in the liver of obese individuals with severe fatty liver disease, namely, with steatohepatitis and/or clinically significant fibrosis. IL32 is an atypical multifaceted cytokine associated with several diseases and inflammatory conditions, mainly expressed by immune cells including natural killer (NK), monocytes and T-cells [15,16,17]. Although nine different isoforms are identified, IL32α, IL32β, IL32γ and IL32δ are the most expressed [18,19]. We showed that IL32 is not only expressed by immune cells, but also by hepatocytes, where it is induced by lipotoxicity, and, notably, by endothelial cells [20]. IL32 hepatic expression correlated with hepatic fat, liver damage/lipotoxicity, activation of inflammatory gene pathways, angiogenesis and fibrosis [20,21]. Furthermore, there was a strong correlation between the hepatic mRNA expression of IL32 with circulating levels of the encoded protein. These findings suggested that IL32 could be a potential non-invasively biomarker for severe fatty liver disease. 

Of note, IL32 has been reported to regulate endothelial cell function and activation, enhancing the expression of the intracellular adhesion molecule-1 (ICAM1) [22] in response to metabolic cues. An unbalanced regulation of the expression of IL32 isoforms by the endothelium has been involved in the pathogenesis of atherosclerosis [23], and in promoting endothelial inflammation and pre-eclampsia in pregnant women [24]. However, studies that directly address the role of IL32 signaling in the pathogenesis of hypertension are lacking.

Here, we hypothesized that increased IL32 production triggered by hepatic fat accumulation and lipotoxicity may lead to endothelial activation and development of hyperntesion in individuals with metabolic risk factors. The aim of this study was, therefore, to examine the relationship between circulating IL32 concentration and blood pressure control in a cohort of individuals with metabolic dysfunction at high risk of MAFLD (the Liver-Bible-2021 cohort) with extensive adjustment for metabolic comorbidities. 

## 2. Results

### 2.1. Clinical Features of the Study Cohort and Circulating IL32 Distribution

The clinical features of the 949 apparently healthy individuals with metabolic dysfunction included in the Liver-Bible-2021 are shown in Table 1. Most were in their sixth decade, and predominantly males (83.4%), with a lesser representation of post-menopausal women. About 50% of participants had fatty liver disease and 55.6% had arterial hypertension, whereas compensated diabetes was relatively infrequent (3.7%). Consistently with the early stage of metabolic disease, only 2.1% of participants had LSM ≥ 8.0 kPa consistent with the presence of advanced liver fibrosis. Expectedly, cardiovascular risk was mostly moderate below 10%, but higher in males than in females.

**Figure 1:**
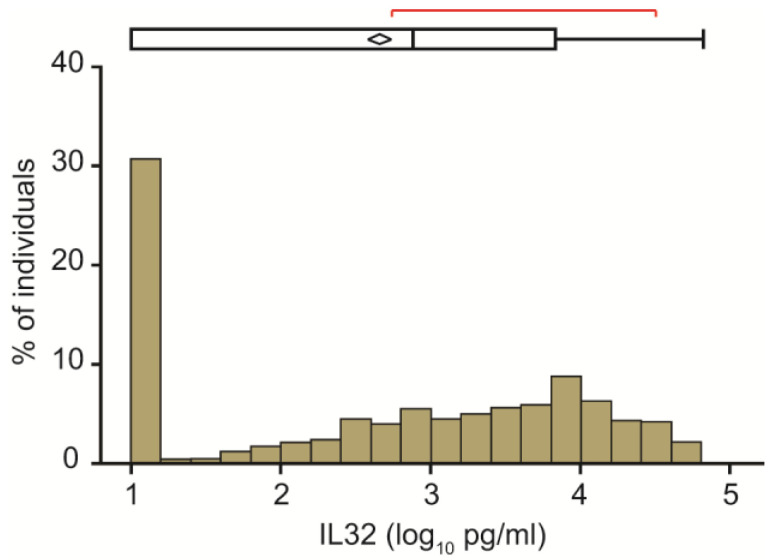
Distribution of circulating IL32 concentration (log_10_ pg/mL) in 949 participants to the Liver-Bible-2021 Cohort.

The distribution of baseline IL32 circulating levels is presented in Figure 1. Median concentration was 2.66 ± 1.28 pg/mL. Remarkably, we detected a high variability in circulating IL32 levels, whose concentration spanned over almost five log_10_ pg/mL among different individuals. In addition, in approximately one third of participants (31.4%) fasting IL32 levels were below the limit of detection (Figure 1).

Although in this cohort characterized by mild liver damage circulating IL32 was not significantly associated with liver stiffness (Table 2), we confirmed that the IL32-ALT-AST score predicted noninvasively assessed fibrosing steatohepatitis (OR 1.30, 95% c.i 1.17–1.43, *p* = 5 × 10^−9^), with moderate accuracy (AUROC 0.75).

### 2.2. Relationship between Circulating IL32 Levels and Metabolic Features

**Figure 2:**
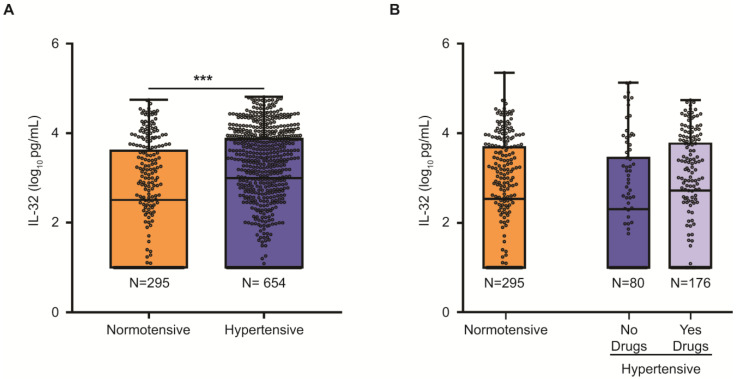
**Panel A** Circulating IL32 (log_10_ pg/mL) in 949 participants to the Liver-Bible-2021 Cohort stratified according to blood pressure control. *** *p* value < 0.001. Error bars = SD. **Panel B**: stratified by use of anti-hypertensive medications (Normal blood pressure, altered blood pressure without medications and with medications).

The relationship between plasma IL32 concentration and metabolic comorbidities is presented in Table 2. Circulating IL32 levels were higher in females (*p* = 0.049) and tended to be associated with a more favorable metabolic profile with lower abdominal circumference (*p* = 0.022), a trend for lower HbA1c (*p* = 0.051) and a Mediterranean dietary pattern (MDS: Mediterranean diet score; *p* = 0.037). However, plasma levels of IL32 showed no association with circulating lipid or insulin levels. Furthermore, IL32 did not correlate with C reactive protein levels in 704 participants where data were available (*p* = 0.26). Impaired blood pressure control was associated with higher IL32 circulating levels (estimate 0.160, 95% c.i. 0.067–0.253, *p* = 0.0007; shown in Table 2 and Figure 2). This association remained significant irrespective of the threshold to define the presence of alteration of blood pressure control (estimate 0.084, 95% c.i. 0.002–0.166, *p* = 0.043 for the presence of hypertension defined according to ESC guidelines). Furthermore, IL32 levels showed a significant association with systolic blood pressure (estimate 0.008, 95% c.i. 0.001–0.015 *p* = 0.017), but not with diastolic blood pressure (0.008, 95% c.i. −0.002–0.019, *p* = 0.11; Table 2).

At multivariable analysis (Table 2, right panel), systolic blood pressure (estimate +0.008 log_10_ per 1 mmHg increase, 95% c.i. 0.002–0.015; *p* = 0.016) and abdominal circumference (estimate −0.009 log_10_ per 1 cm increase, 95% c.i. −0.019–0.000; *p* = 0.040) remained independently associated with circulating IL32. At the same time, both altered blood pressure control (estimate +0.167 log_10_ for those meeting the criteria, 95% c.i. 0.070–0.256; *p* = 0.0006), and hypertension defined according to ESC guidelines (estimate +0.092 log_10_ for those meeting the criteria, 95% c.i. 0.009–0.174; *p* = 0.029) remained independently associated with circulating IL32.

### 2.3. Sensitivity Analyses

When considering IL32 tertiles distribution, in ordinal regression models, blood pressure control (estimate +0.218, 95% c.i. 0.084–0.352; *p* = 0.001) and, to some extent, systolic blood pressure (estimate +0.009, 95% c.i. 0.000–0.019; *p* = 0.06) remained associated with higher levels of IL32.

When limiting the analysis to individuals with detectable circulating IL32, in generalized linear models, blood pressure control (estimate +0.079 log_10_ for those meeting the criteria, 95% c.i. 0.004–0.149; *p* = 0.037) and, to some extent, systolic blood pressure (estimate +0.004 log_10_ per 1 mmHg increase, 95% c.i. −0.001–0.010; *p* = 0.08) remained associated with IL32 levels.

The association of altered blood pressure control with IL32 was present both in participants with and without fatty liver (Table S1). After stratification of participants according to sex, IL32 levels correlated with altered blood pressure in men, whereas the association was not significant in women, who had higher baseline levels, but were less represented in the present cohort (Figure S1A and Table S1). Moreover, in participants reporting a predominantly Mediterranean dietary pattern (Mediterranean Dietary score ≥ 7), the correlation between plasma IL32 levels and altered blood pressure was lost, suggesting a possible modulation of Mediterranean diet on IL32 levels (Figure S1B and Table S1). The impact of individual dietary items evaluated in the Mediterranean Diet Score on circulating IL32 levels are reported in Table S2, showing that the intake of ≥ 1 portions of nuts and ≥ 3 portions of legumes per week were independently associated with higher IL32 (*p* < 0.05).

### 2.4. Impact of Blood Pressure Lowering Medications

To corroborate the association between circulating IL32 levels and altered blood pressure, we analyzed the association between IL32 and the use of anti-hypertensive drugs. Overall, 254 participants were on anti-hypertensive treatments (26.8%, with 38.9% of those with hypertension). Among those meeting the criteria for hypertension at presentation, 120 (12.6%) were on angiotensin converting enzyme inhibitors (ACE-I), 100 (10.5%) on angiotensin receptor blockers (ARB), 56 (5.9%) on diuretics, 49 (5.1%) on calcium antagonists, 31 (3.2%) on beta-blockers and 5 (0.5%) on alpha-blockers. The impact of anti-hypertensive medications on circulating IL32 levels are shown in Table 3. Interestingly, exposure to any antihypertensive therapy showed a protective association with circulating IL32, independently of abdominal circumference and systolic blood pressure (average 0.189 log_10_ decrease in those treated, *p* = 0.0002). Among anti-hypertensive drugs, ACE-I, diuretics and beta-blockers were significantly associated with lower IL32 levels (*p* < 0.05). After adjustment for possible confounders (abdominal circumference and arterial hypertension) only ACE-I and diuretics remained independently associated with lower IL32 (Table 3; *p* < 0.05).

### 2.5. Independent Determinants of Arterial Blood Pressure

The independent determinants of blood pressure levels and control are presented in Table 4. Systolic blood pressure and altered blood pressure control were independently associated with older age, BMI, higher HDL and LDL cholesterol and lack of treatment (*p* < 0.05). Systolic blood pressure as a quantitative trait was also independently associated with glucose control (HbA1c) and alcohol intake. Diastolic blood pressure was associated with BMI glucose control (HbA1c), alcohol intake, cholesterol and alcoholic drinks.

Importantly, higher circulating IL32 concentration was associated with both systolic blood pressure (*p* = 0.010) and altered blood pressure control (*p* = 0.0009) independently of all other covariates. Finally, circulating IL32 tended also to be independently associated with diastolic blood pressure, but the association was not statistically significant (*p* = 0.068).

## 3. Discussion

In this study, we first examined whether circulating levels of IL32, an atypical inflammatory cytokine that is also expressed by endothelial cells, are associated with blood pressure control in a cohort of individuals with metabolic dysfunction from Northern Italy. We were prompted by recent evidence highlighting an association between endothelial and hepatocellular IL32 expression, correlating with circulating levels, with fatty liver disease, insulin resistance and atherosclerosis [20,25]. The underlying hypothesis was that IL32 may bridge hepatic fat accumulation and lipotoxicity with endothelial activation and development of arterial hypertension.

We preliminarily evaluated the distribution of fasting circulating IL32 in the Liver-Bible-2021 cohort of apparently healthy middle-aged individuals with metabolic dysfunction, about two thirds of whom were affected by arterial hypertension. In one third of the cohort, IL32 levels were below the limit of detection. Furthermore, in those with measurable levels, there was a high variability of circulating IL32 levels, spanning almost five log_10_. These data suggest that IL32 is not constitutively secreted in the extracellular space, but released in response to specific forms of tissue injury. Indeed, IL32 contains a weak secretory signal peptide, suggesting that it is released via an unconventional secretory route. One way of non-canonical secretion is through extracellular vesicles [26,27,28]. Indeed, at least some isoforms of IL32 may be released in the extracellular space from apoptotic bodies, or following other forms of cell death [29]. Furthermore, different studies independently reported that IL32 is mainly retained in intracellular compartments, even following induction by cellular stress and inflammation [30,31]. As a consequence, in the present cohort of individuals at low risk of severe liver damage, IL32 performance as a biomarker to discriminate severe fatty liver disease was at best moderate, although we confirmed previous data indicating that a score incorporating IL32 could predict liver damage related to metabolic dysfunction [20]. However, the selection criteria for this cohort differed markedly from previous studies where we found IL32 more strongly associated with the severity of liver damage [20], as healthy blood donors with metabolic dysfunction were enrolled regardless of liver disease. Furthermore, the presence of steatosis and fibrosis was not evaluated by histology, but estimated by transient elastography.

The main study finding was the observation of the association of circulating IL32 with systolic blood pressure and impaired blood pressure control, and the IL32 down-modulation in response to exposure to anti-hypertensive agents. Indeed, fasting plasma IL32 levels were independently associated with impaired blood pressure control, i.e., higher IL32 levels were observed in individuals with BP ≥130/85 mmHg at the time of evaluation in this study, and this finding was confirmed at multiple sensitivity analyses. In this cohort characterized by a high prevalence of hypertension, the association was mainly explained by the link between IL32 and systolic blood pressure levels, but we also observed a non-significant trend for association with diastolic blood pressure levels. Arterial hypertension, defined by the ESC guidelines as systolic office blood pressure ≥ 140 and or diastolic blood pressure ≥ 90 mmHg or blood pressure control through the use of anti-hypertensive medications, was also independently associated with higher levels. These findings are in line with previous reports of a correlation between the circulating levels of pro-inflammatory cytokines released by the liver, namely interleukin-6 (IL-6) with systolic blood pressure, both in healthy subjects and in the setting of chronic inflammatory diseases such as rheumatoid arthritis [32,33], supporting a link between inflammation and hypertension.

IL32 is a multifunctional cytokine with pleiotropic effects and higher circulating IL32 levels were observed in a variety of diseases in which it amplifies inflammatory response, such as rheumatoid arthritis, systemic sclerosis, inflammatory bowel disease and several cancers, but also in coronary artery disease [34,35,36,37]. In this respect, IL32 may play a role in endothelial cell function, and in the activation and proliferation associated wtith vasocontrisction and development of arterial hypertension. Indeed, in patients affected by systemic sclerosis, circulating IL32 levels were higher in those with than those without pulmonary arterial hypertension [38]. In keeping with this, increased IL32 staining was observed in tissue sections from patients with pulmonary hypertension, whereas in cellular models, IL32 silencing decreased endothelial cells proliferation and the expression of the adhesion molecule ICAM-1 [25]. In addition, IL32 was also implicated in the pathogenesis of pre-eclampsia, whose hallmark is the development of arterial hypertension during pregnancy [39,40]. Pulmonary and arterial hypertension share as pathophysiological mechanism endothelial dysfunction, thickening of arterial vessels and smooth muscle cells contraction, as well as high levels of secreted IL32, which have been observed in these pathophysiological conditions. Furthermore, previous studies have already reported an association of IL32 with cardiovascular disease and atherosclerosis. In a Chinese study involving 362 patients with coronary artery disease, IL32 levels correlated with the degree of coronary artery stenosis [41], suggesting the possibility of a pro-atherogenic activity of IL32 and its association with an unstable plaque-phenotype. It can, therefore, be speculated that in individuals with metabolic dysfunction, lipotoxicity represents a trigger leading to increased IL32 transcription and secretion into the bloodstream, and that this process is associated with development of inflammation and activation of capillary endothelial cells leading to vasoconstriction and increased blood pressure levels.

Interestingly, in the present study cohort, the use of anti-hypertensive medications was negatively associated with circulating IL32, and treatment with ACE-I and beta-blockers was associated not only with improved blood pressure control in participants with hypertension, but also with lower levels of IL32. These data suggest that IL32 secretion may reflect endothelial damage and activation. Furthermore, IL32 may be involved in the pathogenesis of hypertension and possibly of the meta-inflammation leading to the progression of cardiovascular disease and its complications. Indeed, a protective anti-inflammatory activity of beta-blockers has previously been reported in patients with cardiovascular disease. In particular, beta-blocker initiation led to a decrease in circulating TNFα in patients with dilated cardiomyopathy [42], whereas a higher dose of ACE-I reduced circulating Interleukin-6 in individuals with chronic congestive heart failure more than a lower dose [43]. On the other hand, these data are also consistent with the alternative hypothesis that IL32 is released in response to altered blood pressure control due to endothelial activation, thereby being potentially implicated in mediating the target organ damage associated with this condition. Further mechanistic and/or genetic studies are therefore required to determine whether higher IL32 levels contribute to the rise in blood pressure or vice versa.

This study has some limitations. First, we measured IL32 at only one single time point for each individual after overnight fasting, and, thus, we could evaluate variations of IL32 that may occur over time and in response to metabolic cues. Furthermore, the diagnostic test employed was designed to detect a common IL32 region without the possibility to discriminate among the various isoforms. These are secreted by different cell types, which could not be assessed in the present study, and may possibly have opposite activity in the regulation of inflammatory pathways by different receptors [20], precluding us to decipher the details of this potential molecular mechanism linking MAFLD and hypertension. We based blood pressure evaluation on a single punctual measurement, whereas the diagnosis of hypertension should rely either on repeated office BP measurements or on out-of-office measurement with ambulatory monitoring and/or home monitoring, if feasible. Finally, results may not be applicable to other ends of the spectrum of metabolic alterations, namely, completely healthy individuals from the general population on one side and patients with severe fatty liver disease and type 2 diabetes on the other side.

In conclusion, our findings suggest that circulating IL32 levels are associated with systolic blood pressure control in individuals with metabolic dysfunction, whereas the use of anti-hypertensive medications reduces IL32. These results suggest that IL32 may be considered as a novel candidate therapeutic target to prevent cardiovascular disease in individuals with metabolic dysfunction. However, further studies are required to confirm these findings and to clarify the mechanism linking IL32 release, inflammation and hypertension.

## 4. Materials and Methods

### 4.1. Study Cohort

The study was conducted in a cohort of apparently healthy individuals with metabolic dysfunction presenting for blood donation from June 2019 to February 2021 at the Transfusion Medicine unit of the Fondazione IRCCS Ca’ Granda Hospital (Liver-Bible-Cohort 2021). Participants aged between 40 and 65 years had to be willing to provide their consent, and have at least three of the following features associated with insulin resistance/metabolic dysfunction: overweight or obesity (Body mass index (BMI) ≥ 25 Kg/m^2^), increased fasting glucose or type 2 diabetes (T2D) (fasting glucose ≥ 100 mg/dL), triglycerides ≥ 150 mg/dL, HDL < 45/55 in M/F, increased blood pressure (BP) values (systolic BP ≥ 130 mmHg and/or diastolic BP ≥ 85 mmHg at blood donation) or being on anti-hypertensive treatment. Part of this cohort has previously been described [44,45]. Individuals with chronic degenerative disorders, except for well controlled arterial hypertension, compensated hypothyroidism and T2D not requiring pharmacological therapy, were excluded from the cohort at first evaluation. Participants completed a self-report survey in which recorded lifestyle and clinical characteristics, including smoking habit, medications and supplement use. Dietary pattern was assessed according to the Mediterranean dietary score [46]. The clinical features of the study cohort are presented in Table 1. Cardiovascular risk at 10 years was estimated according to the “Progetto Cuore” developed in an Italian population [47].

### 4.2. Anthropometric Measurements

Weight and height were measured using standard procedures. Body mass index (BMI) was calculated as weight (kg)/height (m)^2^. Conventional office BP at the time of donation was obtained. Patients remained seated comfortably in a quiet room for 5 min before beginning BP measurement and one BP measurement was recorded. A standard bladder cuff (12–13 cm wide and 35 cm long) was used. The cuff was positioned at the level of the heart, with the back and arm supported to avoid muscle contraction and isometric exercise-dependent increases in BP according to European Society of Cardiology (ESC) 2018 Guidelines [48]. Impaired blood pressure control was defined as increased blood pressure values (≥130 and/or diastolic blood pressure ≥ 85 at blood donation) irrespective of the use of anti-hypertensive medications. The presence of hypertension was defined according to the aforementioned guidelines as systolic BP values ≥ 140 mmHg and/or diastolic BP values ≥ 90 mmHg, or the use of anti-hypertensive medications [48].

### 4.3. Laboratory Analysis

Aminotransferases (ALT, AST), high density lipoprotein (HDL), low density lipoprotein (LDL), cholesterol, triglycerides (TG), glucose and insulin were measured by standard laboratory methods at the Fondazione IRCCS Ca’ Granda core laboratory. The IL32-ALT-AST severe Fatty Liver Disease score was tested for the ability to predict non-invasively assessed fibrosing steatohepatitis [20].

### 4.4. Liver Fat Quantification

Liver stiffness measurement (LSM) and relative controlled attenuation parameter (CAP) were noninvasively assessed by vibration-controlled transient elastography (TE) with FibroScan (Echosens, Paris, France) within 4 weeks of the presentation. According to EASL Clinical Practice Guidelines for non-invasive evaluation of liver disease, the following cut-offs were respectively used to indicate moderate/severe steatosis grade and advanced fibrosis: ≥275 dB/m for CAP (presence of fatty liver) and ≥8.0 kPa for LSM (presence of advanced liver fibrosis). Blood was drawn for evaluation in fasting conditions the morning of TE assessment. Fasting serum levels of alanine and aspartate aminotransferases (ALT and AST) were assessed by the IFCC 37 °C method. Fibrosis-4 (FIB-4), FibroScan-AST (FAST) and Fatty Liver Index (FLI) scores were calculated as previously reported [49,50,51].

### 4.5. Measurement of Circulating IL32

Blood samples were collected after an overnight fasting. For plasma collection, blood was centrifuged at 200× *g* for 15 min and immediately stored at −80 °C at the Fondazione Biological Resource Center (POLI-MI Biobank, which is part of the Italian node of Biobanking and Biomolecular Resources Research Infrastructure, BBMRI). Plasma samples were thawed only once and circulating levels of IL32 were quantified in duplicate using Human IL32 DuoSet ELISA kit (Cat. N° DY3040, R & D Systems, Minneapolis, MN, USA) following the manufacturer’s instructions. The assay is designed to detect IL32α, IL32β and IL32γ with a detection range of 78.5–5000 pg/mL. Samples were diluted 1:2–1:20 in PBS and were measured in duplicate. Analyses were repeated for further dilution or no dilution when values outside scale were detected. Optical density was measured at 450 nm using TECAN infinite F200 PRO instrument (Männedorf, Switzerland). The minimal detectable concentration above blank was 39 pg/mL. When the IL32 concentration was not detectable, it was assumed to be 10 pg/mL [20]. The intra-assay coefficient of variation (CV) was 3.9 ± 5.0%.

### 4.6. Statistical Analysis

For descriptive statistics, categorical variables are shown as number and proportion. Continuous variables are shown as mean and standard deviation (SD) or median and interquartile range (IQR), as appropriate. Variables that were not normally distributed (e.g., IL32) were log-transformed before entering the analyses.

To examine the variables associated with continuous traits, such as circulating IL32 levels, SBP and DBP, we used generalized linear models (GLM). Logistic regression (LR) models were fit to examine binary traits (e.g., variables associated with sex, arterial blood pressure and impaired blood pressure controls). To correct these associations for relevant confounders, we constructed a series of multivariable models adjusted for age, sex, ethnicity and clinical factors significantly associated at univariate analysis (selecting the most robust determinant in each category to avoid collinearity). Complete data were available for all patients included. Data were analyzed using the JMP software version 16 Pro (SAS Institute Inc, Cary, NC, USA). *p*-values < 0.05 were considered statistically significant.

Performance for IL32-ALT-AST score to predicted noninvasively assessed fibrosing steatohepatitis was assessed by the area under the receiver operating characteristic curve (AUROC) in the Liver-Bible-2021 Cohort.

The study design, analyses and results have been reported according to the Strengthening the Reporting of Observational studies in Epidemiology (STROBE) guidelines.

## Figures and Tables

**Table 1:** Clinical features of 949 participants to the Liver-Bible-2021 Cohort (Overall cohort and after stratification according to sex).

	Overall (*n* = 949)	Males (*n* = 791, 83.3%)	Females (*n* = 158, 16.7%)	*p*-Value *
Age, years	53.9 ± 6.4	53.9 ± 6.5	53.6 ± 5.8	0.6
BMI, Kg/m^2^	28.5 ± 3.1	28.5 ± 3.0	28.9 ± 3.5	0.1
Obesity, yes	214 (22.5)	166 (20.9)	48 (30.9)	9.4 × 10^−3^
Diabetes, yes	35 (3.7)	29 (3.7)	6 (3.8)	0.9
ALT, IU/l	30 ± 14.3	31.3 ± 14.2	23.3 ± 12.8	5.6 × 10^−11^
AST, IU/l	24 ± 8	24.5 ± 7.9	21.5 ± 8.3	3.9 × 10^−6^
CAP ≥ 275 dB/m, yes	470 (49.5)	400 (50.6)	70 (44.3)	0.2
LSM ≥ 8.0 kPa, yes	20 (2.1)	20 (2.5)	0	1.0
LDL, mg/dL	123.2 ± 29.4	122.5 ± 28.5	126.4 ± 28.9	0.12
HDL, mg/dL	45.3 ± 10.1	44 ± 9.4	51.8 ± 10.8	6.1 × 10^−16^
TG, mg/dL	168.4 ± 86.5	171.4 ± 90.3	153 ± 62.2	0.014
SBP, mmHg	136.2 ± 12.1	136.0 ± 11.9	137.2 ± 12.6	0.27
DBP, mmHg	85.5 ± 7.9	85.6 ± 7.8	84.7 ± 8.2	0.18
Arterial hypertension, yes	654 (68.9)	552 (69.8)	102 (64.6)	0.22
Smoke, yes	86 (9.1)	71 (9.0)	15 (9.5)	0.85
10-years cardiovascular risk °, %	0.047 ± 0.030	0.053 ± 0.030	0.018 ± 0.010	7.7 × 10^−112^

* At logistic regression models. ° According to “Progetto Cuore” risk calculator developed in the Italian population. (): % values. ALT: alanine aminotransferase; AST: aspartate aminotransferase; LDL: low density lipoprotein cholesterol; HDL: high density lipoprotein cholesterol, TG: triglycerides; SBP: systolic blood pressure; DBP: diastolic blood pressure; CAP: controlled attenuation parameter; LSM: liver stiffness measurement.

**Table 2:** Independent determinants of circulating IL-32 levels (log_10_ pg/mL) in 949 individuals from the Liver-Bible-2021 Cohort.

	Univariable			Multivariable		
	Estimate	95% C.I.	*p*-Value *	Estimate	95% C.I.	*p*-Value **
Age, years	−0.001	−0.014–0.012	0.87			
Sex, F	0.110	0.000–0.219	0.049	0.070	−0.040–0.018	0.22
Abdominal circumference, cm	−0.011	−0.020–−0.001	0.022	−0.009	−0.019–−0.000	0.040
BMI, Kg/m^2^	−0.004	−0.030–0.023	0.079			
Diabetes, yes	−0.159	−0.376–0.058	0.15			
Obesity, Yes	0.013	−0.086–0.111	0.80			
HbA1c, mmol/mol	−0.020	−0.039–0.000	0.051			
Increased blood pressure (≥130/85 mmHg), yes	0.160	0.067–0.253	0.0007	0.167	0.070–0.256	0.0006
Hypertension, yes	0.084	0.002–0.166	0.043	0.092	0.009–0.174	0.029
SBP, mmHg	0.008	0.001–0.015	0.017	0.008	0.002–0.015	0.016
DBP, mmHg	0.008	−0.002–0.019	0.11			
LDL, mg/dL	0.000	−0.004–0.002	0.60			
HDL, mg/dL	−0.190	−0.583–0.203	0.34			
Triglycerides, mg/dL	0.028	−0.153–0.209	0.76			
Insulin, mU/L	0.001	−0.008–0.010	0.76			
Hyperglycemia, Yes	−0.101	−0.271–0.068	0.24			
Log_10_ ALT, IU/l	0.060	−0.136–0.258	0.55			
Log_10_ AST, IU/l	−0.038	−0.219–0.144	0.68			
CAP ≥ 275	−0.023	−0.105–0.058	0.57			
LSM ≥ 7.9	−0.104	−0.412–0.205	0.51			
MDS > 7, Yes	0.090	0.005–0.175	0.037	0.072	−0.013–0.157	0.097
Cardiovascular risk °, %	−1.080	−3.758–1.560	0.47			

* At generalized linear models; ** At multivariable analysis considering as covariates the variables reported in the table. ° According to “Progetto Cuore” risk calculator developed in the Italian population. ALT: alanine aminotransferase; AST: aspartate aminotransferase; LDL: low density lipoprotein cholesterol; HDL: high density lipoprotein cholesterol, TG: triglycerides; HbA1c: glycated hemoglobin; SBP: systolic blood pressure; DBP: diastolic blood pressure; CAP: controlled attenuation parameter; LSM: liver stiffness measurement.

**Table 3:** Impact of use of anti-hypertensive drugs on circulating IL-32 levels.

		Unadjusted			Adjusted		
	Overall (*n* = 949)	Estimate	95% c.i.	*p*-Value	Estimate	95% c.i.	*p*-Value
Any anti-hypertensive, yes	254 (26.7)	−0.162	−0.254–−0.070	0.0005	−0.189	−0.291–−0.088	0.0002
ACE inhibitors, yes	120 (12.6)	−0.137	−0.159–−0.014	0.029	−0.123	−0.247–−0.001	0.049
ARB, yes	100 (10.5)	−0.093	−0.226–0.040	0.17	-	-	-
Beta-blockers, yes	31 (32.6)	−0.233	−0.462–−0.003	0.047	−0.205	−0.462–0.024	0.079
Alpha-blockers, yes	5 (0.005)	−0.157	−0.721–0.408	0.58	-	-	-
Calcium channel blockers, yes	49 (0.05)	−0.076	−0.261–0.109	0.42	-	-	-
Diuretics, yes	56 (0.060)	−0.191	−0.364–0.018	0.031	−0.177	−0.350–0.004	0.044

Generalized linear models unadjusted and adjusted for abdominal circumference (to correct for adiposity and the presence of metabolic risk factors) and presence of arterial hypertension (to correct for the indication for treatment). ACE: angiotensin converting enzyme; ARB: angiotensin receptor blockers. In total, 8.4% of individuals who take anti-hypertensives take more than one type.

**Table 4:** Independent determinants of arterial blood pressure in 949 participants in the Liver-Bible-2021 Cohort.

	SBP				DBP				Impaired Blood Pressure Control			
	Estimate	Lower	Upper	*p*-Value	Estimate	Lower	Upper	*p*-Value	OR	Lower	Upper	*p*-Value
Age, years	0.346	0.226	0.466	2 × 10^−8^	0.006	−0.076	0.088	0.89	1.03	1.01	1.06	0.007
BMI, Kg/m^2^	0.295	0.055	0.536	0.016	0.237	0.072	0.401	0.005	1.05	1.00	1.11	0.053
HDL-C, mg/dL	0.173	0.099	0.248	5 × 10^−6^	0.067	0.016	0.118	0.010	1.04	1.02	1.06	4 × 10^−6^
LDL-C, mg/dL	0.029	0.004	0.054	0.022	0.020	0.003	0.037	0.024	1.006	1.00	1.01	0.031
HbA1c, mmol/mol	0.219	0.036	0.401	0.018	0.040	−0.084	0.165	0.53	1.01	0.97	1.05	0.45
Alcoholic drinks, n/week	0.184	0.058	0.311	0.004	0.102	0.016	0.188	0.020	1.02	0.99	1.04	0.25
Anti-hypertensive drugs, yes	−2.097	−2.949	−1.244	1 × 10^−6^	−0.381	−0.964	0.202	0.20	0.53	0.38	0.75	0.0003
IL-32, log_10_ pg/mL	0.746	0.173	1.318	0.010	0.363	−0.028	0.755	0.068	1.22	1.09	1.38	0.0009

Multivariable generalized (for continuous traits, namely, SBP and DBP) and logistic regression models (for binary traits, namely, impaired blood pressure control) adjusted for covariates presented in the table. SBP: systolic blood pressure; DBP: diastolic blood pressure; LDL-C: low density lipoprotein cholesterol; HDL-C: high density lipoprotein cholesterol.

## Data Availability

The data that support the study findings are available from the corresponding author upon reasonable request.

## Supplementary Materials

The following supporting information can be downloaded at: https://www.mdpi.com/article/10.3390/ijms24087465/s1.
