# Supplementary material for: Circulating Interlukin-32 and Altered Blood Pressure Control in Individuals with Metabolic Dysfunction
Source: Int J Mol Sci. 2023 Apr 18;24(8):7465. doi: 10.3390/ijms24087465 (PMC10138906; doi:10.3390/ijms24087465)
Supplement: Supplementary file 1 [file ijms-24-07465-s001.zip › ijms-2324977-supplementary.pdf]

SUPPLEMENTARY MATERIAL

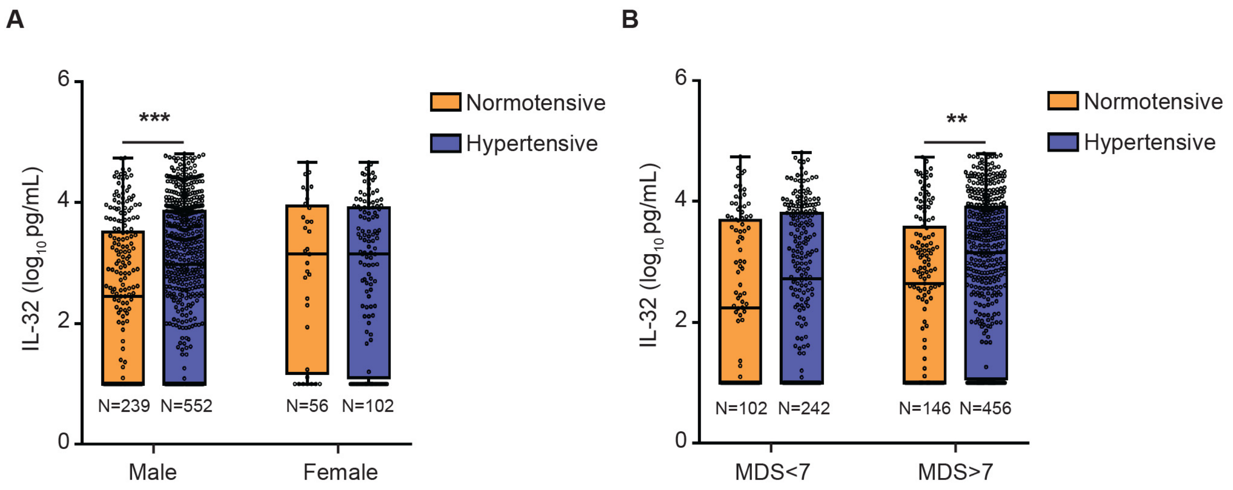

Figure S1. Panel A, B) Circulating IL32 levels gender- and MDS adherence-stratified. IL32 increased significantly in hypertensive male as well as in individuals with low score of Mediterranean diet adherence. P values: **p value <0.01; *** p value <0.001.

SUPPLEMENTARY TABLES

Table S1. Association between IL32 and altered blood pressure controls. stratified for clinically relevant subgroups.

		N=	Estimate	95% c.i.	p-value
FLD	Yes (CAP ≥275 dB/m)	470	+0.141	0.008 – 0.274	0.037
	No (CAP <275 dB/m)	479	+0.190	0.059 – 0.321	0.004
Sex	Male	791	+0.202	0.100 – 0.303	0.0001
	Female	158	-0.001	-0.024 – 0.021	0.91
MDS	>7	595	+0.078	-0.071 – 0.176	0.20
	≤7	354	+0.299	0.152 – 0.446	0.0004

At logistic regression models adjusted for abdominal circumference. FLD: fatty liver disease; MDS: Mediterranean Diet Score.

Table S2. Independent dietary determinants (individual Mediterranean Diet Score items) of circulating IL-32 concentration in 949 participants with metabolic dysfunction in the Liver-Bible-2021 Cohort.

	Estimate	Lower CL	Upper CL	P value
<1 portion of sugar or sparkling drinks / day, No	-0.128	-0.282	0.0267	0.10
<1 portion butter/ day, Yes	0.013	-0.097	0.123	0.81
White meats replace red meats, No	-0.024	-0.107	0.058	0.56
<1 portion of red meat, day, No	-0.064	-0.28	0.154	0.56
<3 sweet servings/ week, No	0.059	-0.029	0.148	0.19
>=3 portions of fruit/ day, No	0.063	-0.029	0.155	0.18
>=3 portions of legumes/ week, Yes	0.102	0.006	0.198	0.038*
>1 serving of nuts/ week, Yes	0.100	0.017	0.184	0.018*
>= 4 tablespoons oil/ day, Yes	0.047	-0.043	0.137	0.30
Olive oil as main fat, No	-0.023	-0.315	0.269	0.87
>=2 simple pasta or rice dishes/ week, No	-0.000	-0.174	0.173	0.99
>=3 portions of fish/ week, No	0.066	-0.041	0.172	0.22
>=2 servings vegetables/ day, No	-0.049	-0.13	0.038	0.27
>= 3 glasses of wine/ week, Yes	-0.071	-0.154	0.011	0.092

At generalized linear models adjusted for each Mediterranean Diet Score item, plus abdominal circumference and systolic blood pressure.
